# Transcriptional changes in mesenteric and subcutaneous adipose tissue from Holstein cows in response to plane of dietary energy

**DOI:** 10.1186/s40104-017-0215-z

**Published:** 2017-12-04

**Authors:** S. J. Moisá, P. Ji, J. K. Drackley, S. L. Rodriguez-Zas, J. J. Loor

**Affiliations:** 1 Department of Animal Sciences, Auburn University, 231 Upchurch Hall, 361 Mell Street, Auburn, AL 36849-5426 USA; 2 Department of Animal Sciences, University of Illinois, Urbana, 61801 USA

**Keywords:** Adipose tissue, Dairy cow, Dietary energy, Transcriptome

## Abstract

**Background:**

Dairy cows can readily overconsume dietary energy during most of the prepartum period, often leading to higher prepartal concentrations of insulin and glucose and excessive body fat deposition. The end result of these physiologic changes is greater adipose tissue lipolysis post-partum coupled with excessive hepatic lipid accumulation and compromised health. Although transcriptional regulation of the adipose response to energy availability is well established in non-ruminants, such regulation in cow adipose tissue depots remains poorly characterized.

**Results:**

Effects of ad-libitum access to high [HIGH; 1.62 Mcal/kg of dry matter (DM)] or adequate (CON; 1.35 Mcal/kg of DM) dietary energy for 8 wk on mesenteric (MAT) and subcutaneous (SAT) adipose tissue transcript profiles were assessed in non-pregnant non-lactating Holstein dairy cows using a 13,000-sequence annotated bovine oligonucleotide microarray. Statistical analysis revealed 409 and 310 differentially expressed genes (DEG) due to tissue and diet. Bioinformatics analysis was conducted using the Dynamic Impact Approach (DIA) with the KEGG pathway database. Compared with SAT, MAT had more active biological processes related to adipose tissue accumulation (adiponectin secretion) and signs of pro-inflammatory processes due to adipose tissue expansion and macrophage infiltration (generation of ceramides). Feeding the HIGH diet led to changes in mRNA expression of genes associated with cell hypertrophy (regucalcin), activation of adipogenesis (phospholipid phosphatase 1), insulin signaling activation (neuraminidase 1) and angiogenesis (semaphorin 4G, plexin B1). Further, inflammation due to HIGH was underscored by mRNA expression changes associated with oxidative stress response (coenzyme Q3, methyltransferase), ceramide synthesis (N-acylsphingosine amidohydrolase 1), and insulin signaling (interferon regulatory factor 1, phosphoinositide-3-kinase regulatory subunit 1, retinoic acid receptor alpha). Activation of ribosome in cows fed HIGH indicated the existence of greater adipocyte growth rate (M-phase phosphoprotein 10, NMD3 ribosome export adaptor).

**Conclusions:**

The data indicate that long-term ad-libitum access to a higher-energy diet led to transcriptional changes in adipose tissue that stimulated hypertrophy and the activity of pathways associated with a slight but chronic inflammatory response. Further studies would be helpful in determining the extent to which mRNA results also occur at the protein level.

**Electronic supplementary material:**

The online version of this article (10.1186/s40104-017-0215-z) contains supplementary material, which is available to authorized users.

## Background

Dairy cows can readily overconsume dietary energy during the prepartum period [1]. Offering a high-energy compared with a low-energy diet leads to increased internal fat deposition [2]. Studies conducted by different research groups revealed that excess prepartal energy intake induced higher prepartal plasma concentrations of insulin, glucose and beta-hydroxy-butyric acid, in comparison with controlled or restricted energy feeding. This symptomatology was associated with greater peripheral lipolysis, with subsequent greater hepatic lipid accumulation at the onset of lactation that compromised animal health [3, 4]. A decrease in adipose tissue (AT) responsiveness to insulin was proposed as the reason for negative effects on animal performance.

Adipose tissue is not simply a metabolic tissue that regulates whole body energy homeostasis, it also plays an important endocrine function by secreting a number of proteins with signaling properties that are involved in the regulation of metabolism (adiponectin, leptin), feed intake (leptin), and immune function and inflammation (TNF-α, IL-1β) [5]. Despite the dominance of mature adipocytes, AT is also composed of immune cells (macrophages) and stromal-vascular cell fractions containing preadipocytes, endothelial cells, and mesenchymal stem cells, which may vary in their response to external stimuli (such as nutrient supply) and immune activation [5].

Differences between visceral AT (VAT) and SAT in the proportion of cell types, capillary network, lipid storage capacity, endocrine activity, and responsiveness to lipolytic stimuli have been documented in humans and rodents [6]. In dairy cattle, VAT is more sensitive to dietary changes and it may have a significant effect on whole body metabolic responses, particularly in the liver, due to the direct portal drainage [7]. Some metabolic disorders that occur frequently after parturition are associated with macrophage infiltration into omental and subcutaneous fat, and coincide with a period of high lipolytic activity [8]. In postpartum heifers, this could happen as early as 1 d after parturition [9]. It is currently unknown if AT macrophage infiltration occurs in dairy cattle only during periods of negative energy balance [10].

Large-scale mRNA expression techniques allow detection of changes in thousands of genes simultaneously, which provides a holistic understanding when adequately compared to other omes. The combined utilization of bioinformatics analysis helps identify the most enriched biological functions, pathways, and physiological changes within the affected genes. As an example, in a previous study in mice utilizing microarray analysis of peri-gonadal AT, adiposity varied due to sex, diet, and obesity-related mutations [11]. Furthermore, phenotypic variation in body mass, adipocyte size, and body mass index (BMI) was correlated with quantitative variations in the expression of genes. In that study, body mass was correlated with 1,304 transcripts, and among the top 100 correlated genes a total of 30% encode macrophage-related proteins. For example, TNF-α, IL-6, PAI-1, NO, factor VII, and MCP-1 were correlated with adverse pathophysiological phenotypes associated with obesity [11]. iNOS and TNF-a were required for obesity-induced insulin resistance in mice. Colony stimulating factor 1 receptor (Csf1r) and CD68 antigen (Cd68) were positively correlated with BMI, and succinate dehydrogenase complex iron sulfur subunit B (Sdhb), and ubiquinol–cytochrome c reductase (Uqcr), negatively correlated with body mass. Clearly, gene transcription is a major control mechanism of AT lipogenesis during early lactation [12]. In the current study, we utilized different bioinformatics tools (IPA, DAVID, and KEGG bioinformatics software plus DIA) to identify the most-enriched gene ontology (GO) functions and pathways in MAT and SAT of multiparous dairy cows in response to ad-libitum access for 8 wk of a high energy as compared to a control diet.

## Methods

### Animals and tissue sample collection

All live-animal experimental procedures were approved by the Institutional Animal Care and Use Committee at the University of Illinois. This study used a subset of 10 cows (5/treatment) from a larger study [2, 7, 13] consisting of 18 non-pregnant and non-lactating Holstein cows (body weight = 656 ± 29 kg) from the University of Illinois Dairy Research Unit. Cows averaged 3.0 parities (range 2 to 4). We used nonpregnant, nonlactating cows to replicate the effects of overfeeding in typical production systems without the confounding hormonal changes that occur around parturition. Cows were blocked by initial BCS and previous experimental treatment and were randomly assigned within block to either a diet containing 1.35 Mcal/kg net energy for lactation (dry matter basis; control group, CON) or 1.62 Mcal/kg (high energy group, HIGH) for 8 wk before slaughter and tissue collection. Nutrient composition of the experimental diets can be found in a previous paper [2]. Samples of subcutaneous and mesenteric AT were harvested immediately post-slaughter and snap-frozen in liquid-N until RNA extraction. The different adipose depots located in the body vary in their impact on metabolic risk due to inherent differences in their metabolism (i.e., SAT is less metabolically active than MAT). Furthermore, dairy cattle accumulate relatively more fat in internal adipose depots and less in subcutaneous fat than do beef cattle [14]. Therefore, two fat depots with different metabolic capacity were chosen for this study.

### RNA extraction

Complete details of these procedures are reported elsewhere [13]. Tissue subsamples (2 g) were transferred to a tube containing ice-cold Trizol (Invitrogen Corp., San Diego, CA) reagent and immediately subjected to RNA extraction as previously described [15]. Genomic DNA was removed from RNA with DNase (Qiagen, Valencia, CA) using RNeasy Mini Kit columns (Qiagen). Integrity of RNA (RIN) was assessed as previously described [9]. The RNA concentration was measured using a NanoDrop ND-1000 spectrophotometer (www.nanodrop.com). The purity of RNA (A_260_/A_280_) was above 1.9.

### Microarray protocol

We used a 13,257 annotated bovine oligonucleotide microarray (Illumina, San Diego, CA) containing >10,000 unique elements, which is publicly accessible in the National Center for Biotechnology Information (NCBI) Gene Expression Omnibus (GEO) database (GSE16426) [16]. Hybridizations were performed in a dye-swap reference design. The reference sample was made by pooling RNA from several bovine adipose tissues (SAT, MAT and omental AT). The cDNA were obtained by reverse transcriptase in a 30-μL reaction containing 8 μg RNA, 2 μL of random hexamer primers (3 μg/μL; Invitrogen Corp., CA), 1 μg oligo dT18 (Operon Biotechnologies, Huntsville, AL), and DNase-RNase-free water to a volume of 17.78 μL. The mixture was incubated at 65 °C for 5 min and kept on ice for 3 min. To the mixture were added 12.2 μL solution composed of 6 μL 5× First-Strand Buffer, 3 μL 0.1 mol/L DTT, 0.6 μL 100 mmol/L dNTP mix (Invitrogen Corp.), 0.12 μL of 50 mmol/L 5-(3-aminoallyl)-dUTP (Ambion, CA), 2 μL (100 U) of SuperScript^TM^ III RT (Invitrogen Corp.), and 0.5 μL of RNase Inhibitor (Promega, Agora, WI). The reaction was performed at 23 °C for 1 min and 46 °C for 9 h. The cDNA obtained was then treated with 10 μL 1 mol/L NaOH and incubated for 15 min at 65 °C to remove residual RNA. The solution was neutralized by adding 10 μL of 1 mol/L HCl. The unincorporated 5-(3-aminoallyl)-dUTP and free amines were removed using a Qiagen PCR Purification Kit (Qiagen). Clean cDNA was dried and re-suspended in 4.5 μL of 0.1 mol/L Na_2_CO_3_ buffer (pH 9.0) and 4.5 μL of Amersham CyDye™ fluorescent dyes diluted in 60 μL of DMSO (Cy3 or Cy5; GE Healthcare, Waukesha, WI, USA). Binding of Cy dyes with 5-(3-aminoallyl)-dUTP incorporated into cDNA was obtained by incubation at room temperature for 1 h. The unbound dyes were removed using a Qiagen PCR Purification Kit (Qiagen) and clean labelled cDNA was measured by means of a NanoDrop ND-1000 spectrophotometer. Sample and reference were then vacuum-dried in the dark.

### Microarray hybridization and image acquisition

Prior to hybridization, slides were re-hydrated, placed in an UV cross-linker, washed with 0.2% SDS solution, thoroughly rinsed with purified water to remove un-bound oligonucleotide, and pre-hybridized using a solution containing 1% albumin, 5 × SCC, and 0.1% SDS at 42 °C for ≥45 min with the aim of decreasing background. After pre-hybridization, slides were rinsed with abundant purified water, immersed in isopropanol for ~10 s, and spin-dried. Dried slides were immediately hybridized in a dye-swap-reference design (i.e., each sample was labeled twice with each of the two dyes and hybridized in each slide with the reference labeled with the opposite dye). Labeled cDNA of the sample was re-hydrated with 80 μL of hybridization buffer #1 (Ambion, Austin, TX) and mixed thoroughly. This solution was used to re-suspend the reference sample labeled with the opposite dye and mixed thoroughly in order to obtain a homogenous solution of the two labeled cDNA. Before hybridization, the labeled cDNA resuspension of the sample plus reference was incubated at 90–95 °C for 3 min to allow for cDNA denaturation to increase the efficiency of binding of oligos to the slide.

Hybridizations were carried out using humidified slide chambers (Corning, Lowell, MA) with cover slips (LifterSlip; Thermo Scientific, Billerica, MA) at 42 °C for 40 h in the dark. After hybridization, slides were removed from the chamber and washed for 5 min by agitation 3 times with wash buffers in the following order: 1 × SSC and 0.2% SDS solution preheated at 42 °C, 0.1 × SSC and 0.2% SDS solution, and 0.1 × SSC solution. Lastly, slides were inserted into a 50-mL tube, spin-dried and gassed with Argon to preserve dye from bleaching. Arrays were scanned with a ScanArray 4000 (GSI-Lumonics, Billerica, MA) dual-laser confocal scanner and images were processed and edited using GenePix 6.0 (Axon Instruments). Array quality was assessed using an in-house parser written in Perl language as previously described [16]. Spots that received a − 100 flag by GenePix 6.0 were removed from further analysis and background intensity was subtracted from the foreground intensity. Spots on the slide were considered ‘good’ if the median intensity was ≥3 × standard deviations above median background for each channel (i.e. dye). Spots were flagged ‘present’ when both dyes passed the criteria, ‘marginal’ if only one dye passed the criteria, or ‘absent’ when both dyes failed to pass the criteria. Statistical analysis was conducted on oligos that were flagged as ‘present’ and ‘marginal’.

### Quantitative PCR for microarray verification

The protocols for cDNA synthesis, real time RT-PCR, primer design, and testing have been previously described [17]. The expression of 29 genes whose protein products are associated with lipid metabolism, inflammation, and insulin signaling were determined through qPCR. Initial PCR data was normalized with the geometric mean of 3 pre-determined internal control genes (*TRIM41*, *KEAP* and *MRP63*). Additional file 1: Table S1 depicts the expression patterns (fold-change) of the 29 genes analyzed by qPCR and microarray. Overall, microarray and qPCR verification results coincide in terms of activation or inhibition with significant differences in fold change.

### Statistical analysis

Animal performance data for cows used in this study was reported in a previous paper [2]. Data from a total of 20 microarrays were normalized for dye and array effects (i.e. Lowess normalization and array centering and scaling) and used for statistical analysis. A mixed effects model was then fitted to the adjusted ratios (adipose/reference) of each oligonucleotide using Proc MIXED (SAS Institute, Inc., Cary, NC, USA). The model consisted of the classification factors tissue (SAT, MAT), diet (CON, HIGH), and dye (Cy3, Cy5) as fixed effects, and cow as a random variable. The statistical model used was: Yijklm = μ + Ti + Dj + Wk + Sl + (T × D)ij + (D × W)jk + (T × W)ik + (D × T × W)ijk + εijklm; where, *Yijklm* is the background-adjusted normalized fold change value; *μ* is the overall mean; *Ti* is the fixed effect of tissue (2 levels); *Dj *is the fixed effect of diet (2 levels); *Wk* is the fixed effect of dye (2 levels); *Sl* is the random effect of cow nested within treatment; T × D, D × W, T × W are the interactions of tissue by diet, diet by dye and tissue by dye, respectively; D × T × W is the interaction of third order for the main effects; and *εijklm *is the random error (0, σ_e_^2^) associated with *Yijklm*. All means were compared using the PDIFF statement of SAS (SAS Institute, Inc., Cary, NC, USA). The mean of the two spots for each oligonucleotide within each array and between dye-swap arrays was not averaged prior to statistical analysis. Benjamini and Hochberg’s false discovery rate (FDR) was used to adjust for the number of comparisons, and significance was declared at FDR ≤ 0.2 (adjusted *P*-value). For qPCR verification, a 2 × 2 factorial arrangement in GLM was used with tissue and diet as the two factors.

### Data mining

The entire microarray data set with associated statistical *P*-values were imported into Ingenuity Pathways Analysis ® (IPA, www.ingenuity.com) in order to examine the number of activated and inhibited DEG. Entrez Gene IDs were used to identify individual sequences. Bioinformatics analysis of microarray data was performed using Dynamic impact approach (DIA) [18] and information from the freely-available online databases Kyoto Encyclopedia of Genes and Genomes (KEGG) and Database for Annotation, Visualization, and Integrated Discovery (DAVID) v6.7. A list of gene identifiers (Entrez Gene IDs) was uploaded all at once to extract and summarize functional annotations (categorized based on Gene Ontology [GO] as biological process [Bp], cellular component [Cc], molecular function [Mf] or interpro [Interpro]) associated with groups of genes or with each individual gene. Details of the DIA approach and its validation have been reported previously [18]. The interpretation of the bioinformatics analysis was performed following the same approach as our previous study [19]. The most impacted pathways (high impact values) were obtained by evaluating those pathways with calculated impact values above 50% of the total impact value within the top-impacted pathways and GO terms (Additional file 2: Figure S1, Figure S2). A visual explanation for interpretation of the Dynamic Impact Approach output could be found in a previous paper [18]. Clearly, by focusing only on the transcriptome at the pathway level, the present study has some limitations as it is unknown whether differences would translate to the protein expression level. A complete table with microarray results for the comparisons MAT vs. SAT and HIGH vs. CON can be found in Additional file 3.

## Results and discussion

### Animal performance and adipose depot response to diets

Animal performance, adipose tissue mass comparisons and blood serum metabolite data from the larger group of 18 cows were reported elsewhere [2]. Briefly, cows fed HIGH consumed 42% more DM than cows fed CON, therefore, final BW was greater (*P* < 0.05) for cows fed HIGH than for those fed CON. However, final BCS and carcass weight did not differ significantly between dietary groups [2]. The concentration of glucose in serum did not differ between diets, but serum insulin tended (*P* = 0.09) to be greater for cows fed HIGH than for those fed CON. As a result, the glucose to insulin ratio was greater (*P* = 0.02) for cows fed CON than for those fed HIGH. Concentrations of NEFA, urea N, total protein, albumin, and globulin did not differ significantly between diets.

Adipose tissue accumulates in different areas in beef and dairy cattle, being usually higher in internal adipose depots for dairy cattle and higher in subcutaneous depots in beef steers [14]. The amount of fat depot in an specific area of the body will also depend on the type of diet provided [20]. In our study, dairy cows fed HIGH had greater mesenteric adipose mass compared to CON cows [2]. In ruminants, this selective deposition of lipid is mainly due to differences in the final products of fermentation by rumen microorganisms. Beef cattle are usually fed high starch-containing diets resulting in more propionate available for metabolism than dairy cattle, which usually receive a forage-based diet where acetate is more predominant than propionate [21]. The glucose converted to propionate by ruminal microbes is the favored substrate for adipocytes infiltrating muscle (i.e., intramuscular fat), unlike subcutaneous adipocytes where acetate is favored as lipogenic substrate [22].

### Transcriptomic modifications in different adipose depots

#### Differentially expressed genes induced by dietary energy in mesenteric (MAT) and subcutaneous adipose (SAT) depots

**Fig. 1:**
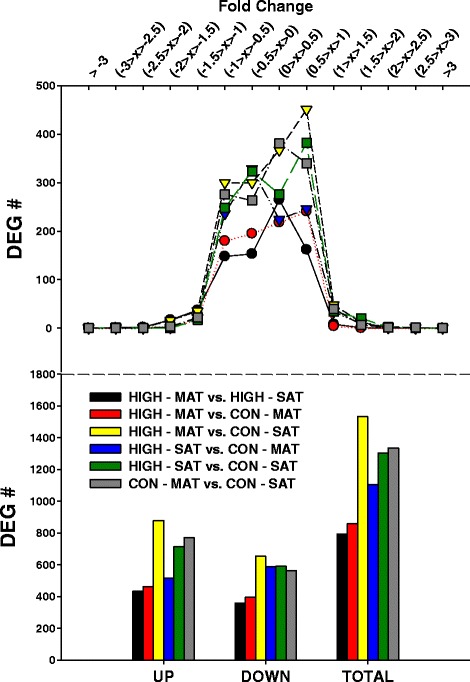
Differentially expressed genes for MAT or SAT fat depots of cows fed HIGH or CON diets. Number of total, up and down differentially expressed genes (DEG; FDR ≤ 0.2) and their expression. Fold-change scale from −3 to 0 for down-regulated and from 0 to 3 for up-regulated DEG across the different comparisons. Data are for effect of different dietary treatments (HIGH and CON) and two different fat depots (MAT and SAT)

In this study, the comparison between MAT of cows fed HIGH with the SAT of cows fed CON had the greatest overall number of DEG, including greater numbers of up-regulated DEG (Fig. 1). The majority of DEG had a range in fold-change of −1.5 and 1-fold with the highest number of DEG for the comparison HIGH-MAT vs. CON-SAT (Fig. 1). In other words, cows that were fed CON had lower overall DEG in SAT suggesting a greater metabolic activity in MAT for cows fed HIGH. It is important to point out that, at a transcriptome level, a higher energy supply in the diet produces activation of genes present in fat depots like SAT which is more sensitive to insulin (antilipolytic stimulation) as compared to MAT which is more sensitive to catecholamines (lipolytic stimulation) [23].

Dry matter intake was greater in non-lactating cows fed the high energy diet as compared to the low energy diet; as a consequence, total visceral and abdominal fat mass was greater in cows fed high energy [2]. Therefore, in HIGH cows, these are signs suggesting that MAT had a more active metabolism with a higher turnover rate as compared with SAT. Hence, we expect that MAT will be more sensitive to dietary changes based on the greater DM and energy intake [2].

#### Most impacted DEG, KEGG pathways and GO terms in MAT and SAT depots

**Fig. 2:**
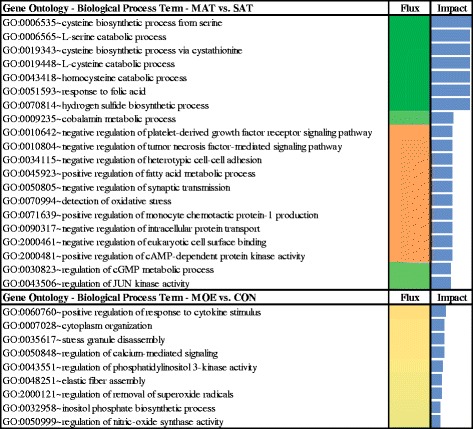
Significant GO Terms (BP) for MAT or SAT fat depots of cows fed HIGH or CON diets. Significant biological processes (BP) among differentially expressed genes (*P* value <0.05; FDR < 0.20) in the comparison of MAT or SAT fat depots between cows fed HIGH or CON diets. Flux represents the direction of each category and the corresponding subcategory (green color = inhibition, yellow color = stable, red color = activation with different color intensities according with the level of up-regulation or down-regulation). Blue bars denote the impact of each BP. Results of flux and impact of the BP with impact value above the 50% of the maximum total impact of each comparison. For complete list of BP see Additional file 2: Figure S1, and Figure S2

The ‘Cysteine and methionine metabolism’ KEGG pathway was highly impacted and inhibited due to the effect on expression of cystathionine-beta-synthase (*CBS*), which leads to homocysteine catabolism inhibition and hydrogen sulfide biosynthesis (Fig. 2 and Additional file 4). The gene *CBS* has a pivotal role in mammalian sulfur metabolism at the homocysteine junction, where methionine is conserved or converted to cysteine via the trans-sulfuration pathway [24].

In ruminant pancreas and liver, *CBS* regulates the conversion of homocysteine to cystathionine, and the conversion of cystathionine to cysteine is catalized by the activity of γ-cystathionase. γ-cystathionase is a rate-limiting enzyme for homocysteine catabolism in sheep, and in adult sheep more homocysteine is conserved for methionine synthesis by lowering the rate of the catabolism of homocysteine to cysteine [25]. Thus, we speculate that in MAT vs. SAT, the marked downregulation of ‘cysteine and methionine metabolism’ pathway might be driven by a similar mechanism. Furthermore, cysteine-related compounds also protect the liver against oxidative stress damage via enhancing glutathione peroxidase (GPx) activity [26]. Thus, *CBS* inhibition in MAT vs. SAT could have decreased the synthesis of two potent antioxidants, taurine or glutathione via the transulfuration pathway [27]. If such response actually occured, taurine or glutathione synthesis would have been reduced causing additional infiltration of adipose tissue by macrophages [28]. The inhibition of ‘cysteine biosynthesis’ due to *CBS* downregulation suggests increased sensitivity to oxidative stress in MAT vs. SAT.

In our previous study, mRNA expression of chemokines and adipokines was greater in MAT as compared to SAT, sustaining the outcomes for this study. In a study with mice, pre-adipocytes from subcutaneous adipose tissue were significantly more resistant than visceral-derived cells to cell death caused by oxidative stress [29]. Furthermore, ‘Glycine, serine and threonine metabolism’ was the KEGG pathway with the greatest impact and was inhibited, mainly due to the expression of alanine-glyoxylate aminotransferase (*AGXT*) that was up-regulated, and *CBS* which was down-regulated. Both genes have a role in L-Serine catabolism leading to the synthesis of ‘Cysteine via cystathionine’, which was inhibited in MAT as compared to SAT (Additinal file 2: Figure S1 and Additional file 4).

The inhibition of ‘protein digestion and absorption’ KEGG pathway was highly impacted due to the inhibition of a wide variety of collagen genes (*COL1A1*, *1A2*, *3A1*, *5A2*, *9A2*) that play key roles in protein heterotrimerization (Additional file 4). Furthermore, the effect of different collagen-related genes that were down-regulated (*COL1A1*, *1A2*, *3A1* and *5A2*) also seemed related to the inhibition of the KEGG pathway ‘ECM-receptor interaction’ (Additional file 4). The DIA analysis results revealed that among the GO terms associated with ‘ECM-receptor interaction’ KEGG pathway, the highest impact and inhibition was on ‘protein heterotrimerization’ and ‘negative regulation of immune response’ (BP), ‘platelet-derived growth factor binding’ and ‘extracellular matrix structural constituent’ (MF), ‘collagen type I trimer’ (CC), ‘Fibrillar collagen’, ‘C-terminal’ (INTERPRO) (Additional file 4). The inhibition of collagen synthesis during negative energy balance decreases the differentiation capacity of bovine intramuscular preadipocytes [30]. In contrast, during states of positive energy balance in mouse, weakening of the extracellular support of adipocytes, which are mainly formed by collagen, enables their stress-free expansion and is associated with the appearance of an inflammatory profile [31]. In a previous transcriptome analysis using RNA-Seq in intramuscular, subcutaneous and omental fat in Hanwoo steers, ECM-receptor interaction was one of the commonly enriched pathways in all three adipose depots under study. The main ECM constituent, collagen was significantly enriched in subcutaneous and intramuscular fat and authors argued that the interaction between ECM components and transmembrane receptors of fat cells might influence the adipogenic capacity of each depot [32]. Based on this literature, the inhibition of protein heterotrimerization which is related to the collagen type I trimer detected in MAT vs. SAT could be considered as an indirect sign of adipose tissue expansion.

‘Vitamin digestion and absorption’ was inhibited due to the down-regulation of ATP-binding cassette, sub-family C (CFTR/MRP), member 1 (*ABCC1*) and transcobalamin II (*TCN2*) likely related to the cobalamin transport process inhibition in MAT (Additional file 4). The inhibition of ‘cobalamin (vitamin B_12_) metabolic process’ in MAT could have been associated with low systemic vitamin B_12_ levels which would partly account for the activation of FA synthesis. However, the fact that the inhibition of ‘cobalamin metabolic process’ in MAT was associated with the vitamin B_12_ transporters *ABCC1* [33] and *TCN2* [34] seems to indicate that uptake of vitamin B12 by MAT could have been a more important factor. In ruminants, vitamin B_12_ is synthesized almost exclusively by ruminal bacteria [35] and is required for the metabolism of propionate and its derivative methylmalonic acid via the TCA cycle [36]. Methylmalonyl CoA is an inhibitor of fatty acid (FA) synthesis in adipose tissue, and it has been proposed that an accumulation of methylmalonic acid may reduce the rate of FA synthesis in ruminant adipose tissue [36].

**Fig. 3:**
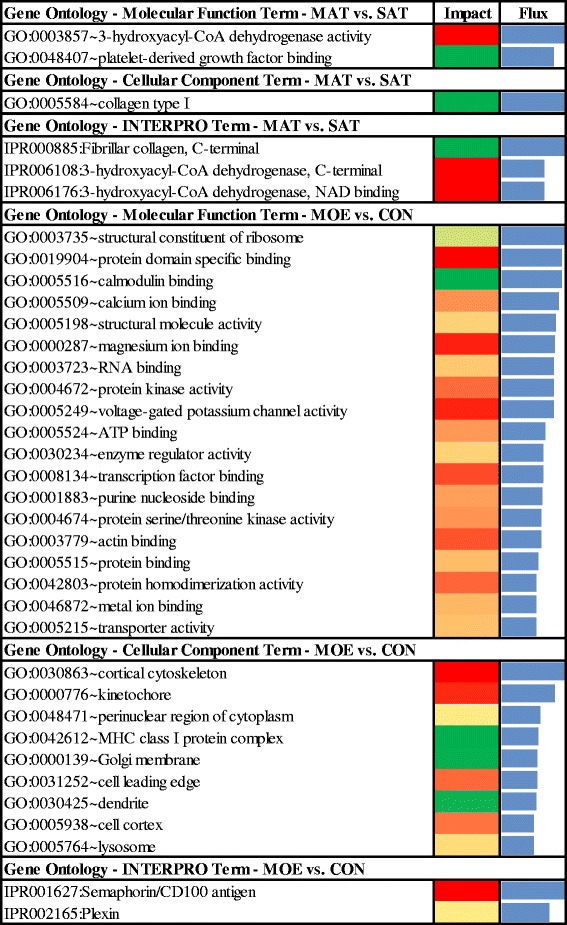
Significant GO terms (MF, CC and INTERPRO) for MAT or SAT fat depots of cows fed HIGH or CON diets. Significant molecular functions (MF), cellular component (CC) and structures inside proteins (INTERPRO) of differentially expressed genes (*P* value <0.05; FDR < 0.20) in the comparison of MAT or SAT fat depots between cows fed HIGH or CON diets. Flux represents the direction of each category and the corresponding subcategory (green color = inhibition, yellow color = stable, red color = activation with different color intensities according with the level of up-regulation or down-regulation). Blue bars denote the impact of each GO Term. Results of flux and impact of the MF, CC or INTERPRO with impact value above the 50% of the maximum total impact of each comparison. For complete list of MF, CC or INTERPRO see Additional file 2: Figure S1, and Figure S2

Butanoate metabolism was activated in MAT due to the up-regulation of acyl-CoA synthetase medium-chain family member 1 (*ACSM1*) and hydroxyacyl-CoA dehydrogenase/3-ketoacyl-CoA thiolase/enoyl-CoA (*HADHA*), both genes with roles in fatty acid beta-oxidation. A greater impact was detected for *HADHA* due to its ‘3-hydroxyacyl-CoA dehydrogenase activity’ (Fig. 3 and Additional file 4). The up-regulation of *HADHA* and *ACSM1* in MAT suggests an activation of mitochondrial beta-oxidation of fatty acids [37]. This activation of fatty acid breakdown in MAT could represent a response of fat cells to control intracellular levels of fatty acids, which may have negative effects on tissue homeostasis [38].

The ‘proximal tubule bicarbonate reclamation’ pathway also had a high impact due to the up-regulation of phosphoenolpyruvate carboxykinase 1 (*PCK1*) and glutamate dehydrogenase 1 (*GLUD1*) (Additional file 4). The up-regulation of *GLUD1* in MAT (a transhydrogenase in the mitochondria) would allow for transfer of hydrogen from NADH produced in the mitochondrial malate dehydrogenase reaction to the NADP produced in the reversal of the mitochondrial isocitrate dehydrogenase step [36]. Furthermore, under lipogenic conditions, the up-regulation of *PCK1* would be linked to synthesis of glycerol-3-phosphate for TAG synthesis through glyceroneogenesis [39]. Both of these responses in MAT compared with SAT support the view of greater lipogenic activity in the former.

In our study, ‘Sphingolipid metabolism’ had a slight inhibition in MAT due to down-regulation of N-acylsphingosine amidohydrolase (acid ceramidase) 1 (*ASAH1*), sialidase 1 (lysosomal sialidase) (*NEU1*) and sphingosine-1-phosphate lyase 1 (*SGPL1*) coupled with the up-regulation of sphingosine kinase 2 (*SPHK2*). These genes play a role in ceramide metabolism, lipid catabolism, fibroblast migration and blood vessel development (Additional file 4). Results from DIA analysis did not reveal a clear picture of what GO term was most impacted in this pathway because no significantly related GO terms had high impact values (Additional file 4). In these Holstein cows under positive energy balance, generation of ceramides in adipose tissue may contribute to inflammation due to the onset of insulin resistance and macrophage accumulation [40], which usually occur when adipocyte accretion exceeds the homeostatic mechanisms of the growing tissue, altering its normal function, due to an increase in fat accumulation. In overweight dairy cows in the transition period, ceramides have been proposed as a potential effectors in the pathological development of insulin resistance [41].

Sphingolipids contribute to the induction of secretion of pro-inflammatory proteins from adipose tissue which is associated to obesity [42]. Sphingolipid metabolism could be activated by several conditions including pro-inflammatory cytokines, growth factors and oxidative stress [43]. Thus, sphingolipids can be considered downstream effectors of obesity-induced inflammation [44]. In the obese adipose tissue, sphingolipid metabolism shifts towards the generation of sphingosine [42]. Not only sphingosine but ceramide and sphingosine-1-P (*S1P*) are involved in multiple signaling pathways that regulate many biological events including the pathogenesis of obesity. Increased ceramide production activates the pro-inflammatory transcription factor kappa-light-chain-enhancer of activated B cells (*NF-κB*) [45], which is an upstream regulator of the secretion of cytokines and chemokines [43]. Ceramide synthesis is mainly regulated by two enzymes: acid ceramidase (*ASAH1*) and ceramide synthase (*CERS*) [46]. Ceramide breakdown into sphingosine and a fatty acid occurs within lysosomes via the enzyme acid ceramidase (*ASAH1*).

Adiponectin (*ADIPOQ*) was highly up-regulated in MAT compared to SAT and seemed associated with several highly-impacted BP (Fig. 3 and Additional file 4). Although, in contrast to our previous DIA analysis in intramuscular fat of beef steers [47], ADIPOQ was not affected by diet [13]. Higher *ADIPOQ* activation in MAT compared with SAT could be taken as a sign of higher fat accumulation in MAT, i.e. fat accumulation and inflammation are linked in this depot.

#### Transcriptomic modifications in adipose induced by a high energy diet

##### Adipose tissue hypertrophy

‘Ascorbate and aldarate metabolism’ was the KEGG pathway with the greatest impact in response to feeding HIGH as compared with CON. This pathway is mainly controlled by regucalcin (*RGN*) which activates the ‘regulation of calcium-mediated signaling’, ‘*L*-ascorbic acid biosynthetic process’ and the ‘positive regulation of ATPase activity’ (Additional file 5).

This calcium-binding protein increases intracellular calcium ion concentration [Ca^2+^]i [48] and contributes to lipid filling and adipocyte hypertrophy by simultaneously stimulating lipogenesis and suppressing lipolysis [49]. Voltage-mediated calcium channel or [Ca^2+^]i receptor stimulation activates the expression of fatty acid synthase (*FASN*), a key enzyme in de novo lipogenesis in adipocytes [49]. Energy-dependent uptake of calcium into adipocyte endoplasmic reticulum is regulated by insulin via the inhibition of the Ca^2+^ − ATPase complex [50], thus, *RGN* up-regulation in HIGH cows might have altered all these mentioned metabolic processes in a way to induce adipocyte hypertrophy.

Phosphatidic acid phosphatase type 2A (*PPAP2A* or *LPP1*) catabolyzes lysophosphatidic acid (*LPA*) in vivo [51]. *LPA* increases preadipocyte proliferation and inhibits adipogenesis via the activation of lysophosphatidic acid receptor 1 (*LPA1*) [52]. *LPA* can be generated during esterification of fatty acids or by the adipose tissue secreted lysophospholipase D known as Autotaxin (*ATX*), whose expression is increased in obese/insulin-resistant states. In a previous study, ATX-knockout mice had higher fat mass and adipocyte size than wildtype [53]. The up-regulation of *PPAP2A* in cows fed HIGH could be taken as an indicator that preadipocyte proliferation might have been reduced via blocking the effect of *LPA* and *ATX*.

##### Adipose tissue inflammation

‘Ubiquinone and other terpenoid-quinone biosynthesis’ KEGG pathway was activated and had a high impact in cows fed HIGH as compared with CON. In this pathway, the up-regulation of coenzyme Q3 methyltransferase (*COQ3*) was responsible for the high impact, but no significant BP, MF or INTERPRO GO terms associated with *COQ3* were detected (Additional file 5). The process of oxidative phosphorylation that occurs in the mitochondrial inner membrane had a strong activation of the Ubiquinone and other terpenoid-quinone biosynthesis in cows fed HIGH which was partly explained by the up-regulation of *COQ3* (Additional file 5). *COQ3* is an S-adenosylmethionine-dependent methyltransferase [54] needed for Coenzyme Q synthesis [55], which helps to cope with oxidative stress due to its anti-inflammatory response and lipid metabolizing effect [56]. Thus, this result suggests that activation of *COQ3* in HIGH cows might have been a response to counteract the existence of an oxidative stress response due to excessive lipogenesis as a result of the higher-energy diet.

‘Sphingolipid metabolism’ KEGG pathway had a high impact with a slight activation in HIGH due to the combination of a strong down-regulation of N-acylsphingosine amidohydrolase (acid ceramidase) 1 (*ASAH1*) and up-regulation of ceramide synthase 5 (*CERS5*), sialidase 1 (lysosomal sialidase) (*NEU1*) and phosphatidic acid phosphatase type 2A (*PPAP2A*). All of these genes can elicit effects on lipid catabolism (Additional file 2: Figure S2 and Additional file 5). The present study suggests that ceramide synthesis is an important process in MAT and SAT, and was activated with high-impact in cows fed HIGH. Thus, we speculate that this pathway reflects adipose tissue inflammation status as a result of a high energy diet.

‘Ether lipid metabolism’ had a slight activation in MAT resulting from up-regulation of platelet-activating factor acetylhydrolase 1b, regulatory subunit 1 (45 kDa) (*PAFAH1B1*) and phosphatidic acid phosphatase type 2A (*PPAP2A*), with a likely effect on ‘platelet activating factor metabolism’ and ‘protein dephosphorylation’ (Additional file 5). As adipocyte hyperplasia and hypertrophy progress, macrophages and neutrophils infiltrate into the expanding adipose tissue. Platelet-activating factor (*PAF*) mediates vascular permeability and stimulation of inflammatory cells, including platelets and neutrophils, promotion of leukocyte chemotaxis and synthesis of pro-inflammatory mediators (TNF-α, IL-1ß, and IL-6) [57, 58]. The PAF acetylhydrolase (*PAFAH1B1*) inactivates PAF [59], resulting in lyso-PAFs as end product of the reaction [60]. Thus, we speculate that the up-regulation of *PAFAH1B1* in HIGH cows was a response to potentially control macrophage infiltration due to *PAF* inactivation by *PAFAN1B1*.

Activin A exerts pro-inflammatory activity which results in the formation of prostanoids, nitric oxide and cytokines in rat bone marrow derived macrophages [61]. Activin collaborates in the production of inflammatory mediators, including *IL-1ß*, *TNF-α*, *IL-6* and *NOS*, thereby promoting the onset of the inflammatory response [62]. The regulation of nitric-oxide synthase (*NOS*) activity can be controlled via signaling through the Activin A receptor type IIA (*ACVR2A*), which was up-regulated in cows fed HIGH. Activation of *NOS* enhances blood flow to insulin-sensitive tissues and its activity is impaired in insulin resistance [63], a condition that is expected to occur in the HIGH cows. Thus, it is likely that *ACVR2A* exerts its pro-inflammatory role in HIGH cows by up-regulating nitric-oxide synthase (Additional file 5).

##### Ribosome biogenesis

**Fig. 4:**
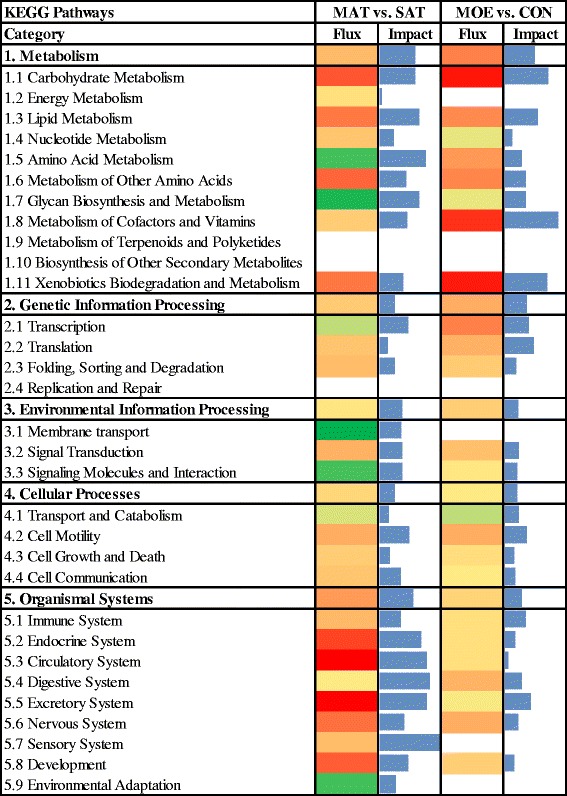
Significant KEGG pathways for MAT or SAT fat depots of cows fed HIGH or CON diets. Analysis performed using the Dynamic Impact Approach (DIA) with differentially expressed genes (*P* value <0.05; FDR < 0.20) using the Kyoto Encyclopedia of Genes and Genomes (KEGG) Pathways database. Flux represents the direction of each category and the corresponding subcategory (green color = inhibition, yellow color = stable, red color = activation with different color intensities according with the level of up-regulation or down-regulation). Blue bars denote the impact of each biological process. MAT: mesenteric adipose tissue; SAT: subcutaneous adipose tissue; HIGH: high energy diet; CON: controlled energy diet

‘Ribosome biogenesis in eukaryotes’ was activated with high impact due to the up-regulation of M-phase phosphoprotein 10 (U3 small nucleolar ribonucleoprotein) (*MPHOSPH10*) and ribonuclease P/MRP 30 kDa subunit (*RPP30*), and the down-regulation of NMD3 ribosome export adaptor (*NMD3*). These genes have a role in ‘RNA splicing’, ‘protein binding’ and ‘protein transport’, respectively (Additional file 5). In contrast, the ribosome pathway was affected by the overall inhibition of the structural constituent of ribosome MF (Fig. 4) due to the up-regulation of mitochondrial ribosomal protein L34 (*MRPL34*), ribosomal protein L21 (*RPL21*), and the down-regulation of ribosomal protein S2 (*RPS2*) and NMD3 ribosome export adaptor (*NMD3*) (Additional file 5).

The biogenesis of both ribosomal subunits (40S and 60S) is a process that begins with the transcription of 35S pre-rRNA by RNA polymerase I in the nucleolus [64]. The 5′ processing factors associate co- and post-transcriptionally with U3 snoRNA to form the U3 snoRNP–pre-rRNA complex which is required for the critical cleavage of rRNA. The protein encoded by *MPHOSPH10* is thought to be part of the U3 snoRNP–pre-rRNA complex, which is involved in nucleolar processing of pre-18S ribosomal RNA [65]. The 35S pre-RNA undergoes a series of cleavage steps required to produce 18S, 25S, and 5.8S rRNA present in the mature ribosome [64]. The U3 snoRNP–pre-rRNA complexes are dynamic entities that begin as small knobs and by condensation become larger knobs as more factors join the complex and the rRNA changes to a more compact conformational structure. The last endonucleolytic cleavage is carried out by the endonuclease activity of the RNase MRP ribonucleoprotein complex (i.e. *RPP30*). Pre-60S ribosomes receive a nuclear export signal from the NMD3 ribosome export adaptor (*NMD3*) and is released from the nucleolus together with U3 snoRNA [66]. Subsequently, the 60S subunit will undergo further maturation steps in the cytoplasm during the process of ribosome biosynthesis [67]. Based on the metabolic processes described above, our transcriptome analysis suggested that ribosome biogenesis was more active in HIGH and might have been associated with the greater adipose tissue mass [2].

##### Transcription factors and inflammation in adipose tissue

The BP ‘positive regulation of response to cytokine stimulus’ had an extremely high impact and was activated due to the up-regulation of TAF9 RNA polymerase II, TATA box binding protein (TBP)-associated factor, 32 kDa (*TAF9*) (Additional file 5). TAFs are components of the transcription factor IID (TFIID) complex, which are essential for the initiation of transcription by RNA polymerase II and the PCAF histone acetylase complex and the STAGA transcription coactivator-HAT complex which are related to epigenetic regulation [68]. *TFIID* is composed of the TATA-binding protein (TBP) and a group of proteins known as TBP-associated factors (TAFs) which are considered co-activators [68]. More specifically, *TAF9* C-terminal region is a critical domain required for transcriptional activation because it is the nexus needed for the recruitment of the *TFIID* complex [68]. The TBP-associated factor 9 (*TAF9*) binds to the basal transcription factor *GTF2B*, which our previous data suggested is responsive to intracellular acetate concentrations within the intramuscular fat particularly when animals are fed higher-fermentable diets [69]. The recruitment of the *TFIID* complex containing TAFs to the Tumor Necrosis Factor, Alpha-Induced Protein 3 (*TNFAIP3*) core promoter may involve a direct interaction between the different TAFs and the specificity protein 1 (*SP1*) transcription factor which regulates the recruitment of the general transcriptional machinery. Furthermore, *TNFAIP3* is activated by *NF-κB* in response to pro-inflammatory signals such as TNF-α and can inhibit inflammation and programmed cell death. As such, this mechanism can reduce damage that inflammation may elicit on tissues [70]. Based on these studies we speculate that, as a component of the *TFIID* complex, *TAF9* has a role in the regulation of the adipose tissue response to cytokines, a process that was highly activated in cows fed HIGH. Because the information available for a clear role of *TAF9* in adipose tissue inflammation is scarce, it appears reasonable to suggest that this TF should be studied further.

Nuclear factor of activated T-cells (*NFAT*) are transcription factors activated by cytosolic calcium, and play an important role in the activation of adipokine expression, differentiation of immune cells, hence, indirectly they contribute to glucose and insulin homeostasis [71, 72]. Transcriptional repression of nuclear factor of activated T-cells, cytoplasmic, calcineurin-dependent 4 (*NFATC4*) by Lipin 1 gene prevents obesity-induced fat tissue inflammation and subsequent insulin resistance [73]. Along with other data indicating an inflammatory and oxidative stress status in fat from cows fed HIGH, we speculate that the up-regulation of *NFATC3* in adipose tissue may have a mechanistic role in the control of inflammation. For instance, it has been reported that *NFATC3* regulates the expression of NOS in macrophages when exposed to LPS [74], resulting in an overall decrease in nitric oxide release and an ensuing reduction in bactericidal activity.

##### Insulin signaling

A strong activation of the pathway ‘regulation of phosphatidylinositol 3-kinase activity’ was mainly due to the up-regulation of phosphoinositide-3-kinase, regulatory subunit 1 (alpha) (*PIK3R1*) and the slight down-regulation of retinoic acid receptor, alpha (*RARA*). Furthermore, the high impact of the pathway ‘regulation of nitric-oxide synthase activity’ was a result of the up-regulation of activin A receptor, type IIA (*ACVR2A*) (Additional file 5). In addition, the up-regulated expression of cytochrome P450, family 2, subfamily S, polypeptide 1 (*CYP2S1*) was responsible for the high impact and activation of the pathway ‘metabolism of xenobiotics’.

Insulin signaling can activate interferon regulatory factor 1 (*IRF1*) by binding to its receptor, leading to recruitment of the regulatory subunit alpha of phosphoinositide-3-kinase (*PIK3R1*). Peroxisome proliferator-activated receptor gamma (*PPARG*) which is PI3K/AKT protein-dependent in adipocytes [75], directly regulates *PIK3R1* expression in adipocytes [76]. Thus, we speculate that the marked activation of ‘regulation of phosphatidylinositol 3-kinase activity’ in cows fed HIGH (Additional file 5) was largely due to these mechanisms of adipogenic transcriptional regulation. It is noteworthy, however, that the DIA analysis also relates *PIK3R1* with Nuclear factor of activated T-cells (*NFAT*) protein import into nucleus and the growth hormone receptor signaling pathway. The connection between *PI3KR1* and *NFAT* protein import into nucleus (Additional file 5) is due to *NFAT* activation through the lipid kinase activity in the Ras/PKC pathway of *PI3K* regulatory subunit [77]. The *PI3KR1* protein participates in the growth hormone receptor signaling pathway and mediates the negative effects of GH on insulin signaling in both skeletal muscle and adipose tissue of dairy cows [78].

The lysosomal enzyme that cleaves terminal sialic acid residues from substrates such as glycoproteins and glycolipids was activated in HIGH cows. The sialic acid residues released by neuraminidase 1 (*NEU1*) activate the N-linked glycan chains of insulin receptor kinase (*IRK*), controlling the onset of insulin resistance [79]. Neuraminidase 1 interacts with *IRK* upon insulin binding to INSR and activates the receptor providing a positive feedback for glucose uptake. The tendency for higher insulin concentrations in cows fed HIGH (*P* < 0.09) [2] likely induced the up-regulation of *NEU1*, and contribute to the increase in fat deposition.

##### Angiogenesis

Within the CC GO terms with high impact due to feeding HIGH, the terms ‘cortical cytoskeleton’ and ‘kinetochore’ were the most impacted. When interior structures within proteins were considered (INTERPRO GO term), Semaphorin/CD100 antigen and Plexin were the most impacted (Additional file 5). Sema domains, present in Semaphorins, had a high impact and was activated due to up-regulation of Semaphorin-4G (*SEMA4G*) and *MET* proto-oncogene, receptor tyrosine kinase (*MET*) (Additional file 5).

Semaphorins and their receptor plexins are cell surface proteins that regulate cell motility in many cell types and elicit a potent pro-angiogenic response [80, 81]. Angiogenesis is a process triggered by adipose tissue hyperplasia and macrophage infiltration [82–84]. Semaphorin 4D–stimulated cell migration requires the activation of the phosphatidylinositol 3-kinase (*PI3K*)-Akt pathway [81] which has a critical role in regulating diverse cellular functions including metabolism, growth, proliferation, survival, transcription and protein synthesis. The binding of Sema 4D (*SEMA4D*) to Plexin B1 (*PLXNB1*) stimulates tyrosine kinase activity of MET proto-oncogene, receptor tyrosine kinase (MET) resulting in tyrosine phosphorylation of both receptors (87). Sema 4D induces angiogenesis through MET recruitment by means of *PLXNB1* (88). As a whole, activation of the mentioned above genes in cows fed HIGH could be taken as an indication of vascular infiltration in adipose tissue as a way to account for the greater drive to deposit lipid (i.e. increase fat mass). Furthermore, the interaction with high specificity of the cytosolic domain of *PLXNB1* in a GTP- and semaphorin CD100 (SEMA4G)-dependent manner with an activated Rac leads to reorganization of actin cytoskeletal structure [85] that will induce cell migration. In our previous study, activation of actin cytoskeleton occurs due to macrophage infiltration in adipose tissue of beef steers [47]. The clear sign of inflammation in our HIGH cows adipose tissue, allow us to suggest that a similar mechanism is activated through the upstream regulation of Semaphorins and Plexins. Lastly, the pro-angiogenic response to HIGH is further supported by the up-regulation of Cytochrome P450 (CYP) epoxygenase (*CYP2S1*), which in non-ruminants has a role in vascular homeostasis [86].

All these changes revealed by the DIA analysis agree with the more pronounced changes in mesenteric fat than in subcutaneous fat when cows are allowed free-access to a high-energy diet [2]. Furthermore, the greater energy intake by HIGH cows would have resulted in greater propionic acid fermentation which stimulates insulin secretion [87]. Therefore, this greater energy intake could have led to the activation of the insulin signaling pathway subsequently enhancing the biological mechanisms leading to adipocyte differentiation, hypertrophy and hyperplasia. The end results of those processes were greater fat mass and likely macrophage infiltration, all of which induced a slight but chronic inflammatory state in the cows.

## Conclusions

The bioinformatics analysis of the transcriptome data from Holstein dairy cows indicated that the mesenteric fat compared with subcutaneous fat has a higher response to level of dietary energy. This was discerned from the activation of differentially expressed genes related to biologic processes including oxidative stress, mitochondrial beta-oxidation, lipogenesis, insulin resistance and macrophage accumulation, together with a greater degree of fat mass accumulation. The effect of chronic ad-libitum access to the high energy diet on adipose tissue was associated with a slight inflammatory response namely due to activation of sphingolipid metabolism. Based on previous work in non-ruminants, we speculate that changes in transcriptomics data pertaining to metabolic processes could translate to the protein level. Hence, they help understand the differences in adipose mass and the responses to high dietary energy reported in these non-pregnant, non-lactating Holstein dairy cows.

## Additional files

Additional file 1: Table S1. Fold change consistency of genes investigated in qPCR and microarray. (DOCX 16 kb)

Additional file 2: Figure S1. Significant KEGG pathways, biological processes (BP), molecular functions (MF), cellular component (CC) and structures inside proteins (INTERPRO) of differentially expressed genes (*P* value <0.05; FDR < 0.20) in the comparison of MAT or SAT fat depots between cows disregarding HIGH or CON diets. Flux represents the direction of each category and the corresponding subcategory (green color = inhibition, yellow color = stable, red color = activation with different color intensities according with the level of up-regulation or down-regulation). Blue bars denote the impact of each BP. Horizontal black lines represent the 50% of the maximum total impact cutoff applied for KEGG pathways or each GO Term. **Figure S2.** Significant KEGG pathways, biological processes (BP), molecular functions (MF), cellular component (CC) and structures inside proteins (INTERPRO) of differentially expressed genes (*P* value <0.05; FDR < 0.20) in the comparison of HIGH vs. CON diets fed to cows disregarding MAT or SAT fat depots. Flux represents the direction of each category and the corresponding subcategory (green color = inhibition, yellow color = stable, red color = activation with different color intensities according with the level of up-regulation or down-regulation). Blue bars denote the impact of each BP. Horizontal black lines represent the 50% of the maximum total impact cutoff applied for KEGG pathways or each GO Term. (XLSX 155 kb)

Additional file 3: Entrez gene ID, gene symbol, fold change, false discovery rate (FDR) and *P* values of the oligonucleotides considered in the microarray analysis for the comparisons of MAT vs. SAT and HIGH vs. CON.. (XLSX 121 kb)

Additional file 4: Integration of significant KEGG pathways, biological processes (BP), molecular functions (MF), cellular component (CC) and structures inside proteins (INTERPRO) related to differentially expressed genes (*P* value <0.05; FDR < 0.20) in the comparison of MAT vs. SAT fat depots of cows disregarding HIGH or CON diets. Flux represents the direction of each category and the corresponding subcategory (green color = inhibition, yellow color = stable, red color = activation with different color intensities according with the level of up-regulation or down-regulation). Blue bars denote the impact of each KEGG pathway or GO Term. (XLSX 68 kb)

Additional file 5: Integration of significant KEGG pathways, biological processes (BP), molecular functions (MF), cellular component (CC) and structures inside proteins (INTERPRO) related to differentially expressed genes (*P* value <0.05; FDR < 0.20) in the comparison of HIGH vs. CON diets received by cows disregarding MAT or SAT fat depots. Flux represents the direction of each category and the corresponding subcategory (green color = inhibition, yellow color = stable, red color = activation with different color intensities according with the level of up-regulation or down-regulation). Blue bars denote the impact of each KEGG pathway or GO Term. (XLSX 1623 kb)

## Abbreviations

ATP: Adenosine triphosphate
BP: Biological process
CC: Cellular component
cDNA: Complementary DNA
CON: Control diet
DEG: Differentially expressed genes
DIA: Dynamic Impact Approach
DM: Dry matter
DMI: Dry matter intake
DNA: Deoxyribonucleic acid
FA: Fatty acid
FDR: False discovery rate
GEO: Gene Expression Omnibus
GO: Gene Ontology
HIGH: High energy diet
INTERPRO: Interior of proteins
KEGG: Kyoto Encyclopedia of Genes and Genomes
MAT: Mesenteric adipose tissue
MF: Molecular function
NCBI: National Center for Biotechnology Information
qPCR: quantitative PCR
RIN: RNA integrity number
RNA: Ribonucleic acid
RT-PCR: Real time polymerase chain reaction
SAT: Subcutaneous adipose tissue
SCC: Saline-sodium citrate
SDS: Sodium dodecyl sulfate
VAT: Visceral adipose tissue
