# Supplementary material for: Transcriptional changes in mesenteric and subcutaneous adipose tissue from Holstein cows in response to plane of dietary energy
Source: J Anim Sci Biotechnol. 2017 Dec 4;8:85. doi: 10.1186/s40104-017-0215-z (PMC5713657; doi:10.1186/s40104-017-0215-z)
Supplement: Supplementary file 1 — Fold change consistency of genes investigated in qPCR and microarray. (DOCX 16 kb) [file 40104_2017_215_MOESM1_ESM.docx]

**Additional Table 1.** Fold change consistency of genes investigated in qPCR and microarray

| Gene | Description | | Fold-Difference | | | | | |
| --- | --- | --- | --- | --- | --- | --- | --- | --- |
|  |  |  | MAT vs SAT | |  | | HIGH vs CON | |
|  |  |  | Microarray | qPCR | Microarray | | | qPCR |
| *Lipid metabolism, fatty acid import* | | | | | | | | |
| *ACLY* | ATP citrate lyase | 1.11 | | 1.52^*^ | | 1.18 | | 1.71^**^ |
| *ACACA* | Acetyl-Coenzyme A carboxylase alpha | -1.09 | | -1.50^*^ | | 1.51 | | 1.52^*^ |
| *FASN* | Fatty acid synthase | -1.10 | | -1.67^**^ | | 1.90 | | 2.10^**^ |
| *ELOVL6* | Elongation of long chain fatty acids | -1.08 | | 1.17 | | 1.55 | | 1.78^**^ |
| *SCD* | Stearoyl-CoA desaturase | -1.60 | | -2.72^**^ | | 4.36 | | 3.50^**^ |
| *GPAM* | Glycerol-3-phosphate acyltransferase | 1.80 | | 1.63^**^ | | 1.48 | | 1.33^*^ |
| *LPL* | Lipoprotein lipase | 1.57 | | 1.65^**^ | | -1.09 | | 1.04 |
| *CD36* | Thrombospondin receptor | 1.31 | | -1.04 | | -1.12 | | -1.01 |
| *ACSL1* | Acyl-CoA synthetase long-chain | 1.38 | | 1.55^*^ | | -1.27 | | -1.09 |
| *ADFP* | Adipose differentiation-related protein | -1.06 | | -1.60^**^ | | 1.22 | | 1.13 |
| *LPIN1* | Lipin 1 | 1.72 | | 2.55^**^ | | 1.09 | | 1.79^**^ |
| *LIPE* | Hormone-sensitive lipase | 1.50 | | 1.04 | | 1.17 | | -1.04 |
| *LEP* | Leptin | 1.02 | | 1.78^**^ | | 1.14 | | 1.39 |
| *ADIPOQ* | Adiponectin | 3.23 | | 1.82^**^ | | -1.06 | | -1.01 |
| *Transcription regulation* | | | | | | | | |
| *THRSP* | Thyroid hormone responsive SPOT 14 | | 2.02 | 1.46^*^ | | 2.53 | | 2.20^**^ |
| *PPARG* | Peroxisome proliferator-activated receptor gamma | | 1.21 | 1.13 | | 1.19 | | 1.15 |
| *SREBF1* | Sterol regulatory element binding transcription factor 1 | | 1.13 | -1.04 | | -1.07 | | -1.41^**^ |
| *MLXIPL* | MLX interacting protein-like | | 1.01 | 1.08 | | 1.07 | | 1.11 |
| *NR2F2* | Nuclear receptor subfamily 2, group F, member 2 | | 1.08 | 1.40^**^ | | -1.20 | | -1.08 |
| *Immune response, acute-phase response, inflammation and insulin sensitivity* | | | | | | | | |
| *TNF* | Tumor necrosis factor | -1.11 | | -1.15 | | -1.52 | | -1.05 |
| *IL-1B* | Interleukin-1 beta | 1.04 | | 1.87^**^ | | 1.15 | | 1.33 |
| *IL-6* | Interleukin-6 | -1.11 | | 1.20 | | -1.13 | | 1.02 |
| *IL-6R* | Interleukin-6 receptor | 1.21 | | 1.38^**^ | | -1.08 | | -1.36^**^ |
| *AKT2* | Thymoma viral proto-oncogene | -1.18 | | 1.25^*^ | | -1.09 | | -1.27^**^ |
| *SAA3* | Acute-phase serum amyloid A1 | -1.29 | | 5.45^**^ | | 1.07 | | -1.48 |
| *TLR4* | Toll-like receptor 4 | -1.03 | | 1.22 | | -1.20 | | -1.18 |
| *CCL2* | Chemokine (C-C motif) ligand 2 | 1.22 | | 1.83 | | 1.09 | | 1.78^*^ |
| *CCL5* | Chemokine C-C motif ligand 5 | -1.07 | | 1.51 | | -1.03 | | 1.08 |
| *INSR* | Insulin receptor | 1.21 | | 1.22 | | 1.02 | | 1.01 |

^**^ *P* ≤ 0.05; ^*^ *P* ≤ 0.10
